# Retirement and Socioeconomic Differences in Diurnal Cortisol: Longitudinal Evidence From a Cohort of British Civil Servants

**DOI:** 10.1093/geronb/gbx058

**Published:** 2017-05-05

**Authors:** Tarani Chandola, Patrick Rouxel, Michael G Marmot, Meena Kumari

**Affiliations:** 1 Cathie Marsh Institute and Social Statistics, University of Manchester, Humanities Bridgeford Street, UK; 2 UCL Eastman Dental Institute and UCL Institute of Education, London, UK; 3 UCL Research Department of Epidemiology and Public Health, London, UK; 4 Institute of Social & Economic Research, University of Essex Wivenhoe Park, Colchester, Essex, UK

**Keywords:** Inequalities, Later Life employment, Stress biomarkers

## Abstract

**Objectives:**

Early old age and the period around retirement are associated with a widening in socioeconomic inequalities in health. There are few studies that address the stress-biological factors related to this widening. This study examined whether retirement is associated with more advantageous (steeper) diurnal cortisol profiles, and differences in this association by occupational grade.

**Method:**

Data from the 7th (2002–2004), 8th (2006), and 9th (2007–09) phases of the London-based Whitehall II civil servants study were analysed. Thousand hundred and forty three respondents who were employed at phase 8 (mean age 59.9 years) and who had salivary cortisol measured from five samples collected across the day at phases 7 and 9 were analysed.

**Results:**

Retirement was associated with steeper diurnal slopes compared to those who remained in work. Employees in the lowest grades had flatter diurnal cortisol slopes compared to those in the highest grades. Low-grade retirees in particular had flatter diurnal slopes compared to high-grade retirees.

**Discussion:**

Socioeconomic differences in a biomarker associated with stress increase, rather than decrease, around the retirement period. These biological differences associated with transitions into retirement for different occupational groups may partly explain the pattern of widening social inequalities in health in early old age.

The socioeconomic gradient in health is well known, although the magnitude of the gradient varies over the lifecourse (House et al., 1994). This socioeconomic-health gradient peaks around retirement in the United States (Deaton & Paxson, 1998; Elo & Preston, 1996; Smith & Kington, 1997) and a number of European countries (Majer, Nusselder, Mackenbach, & Kunst, 2011; van Kippersluis, Van Ourti, O’Donnell, & van Doorslaer, 2009; Van Ourti, 2003) with some evidence of widening even after retirement in the United Kingdom (Benzeval, Green, & Leyland, 2011; Chandola, Ferrie, Sacker, & Marmot, 2007). This widening in health inequalities could be a reflection of the accumulation of socioeconomic disadvantages over the lifecourse with early life inequalities in health becoming magnified over the life cycle.

Retirement could potentially moderate this pattern of widening health inequalities with age if the health effects of retirement differ between socioeconomic groups. Involuntary retirement, which is more common among disadvantaged workers, is associated with poorer health, however, evidence for the effect of overall retirement on health is ambiguous (Banks, Chandola, & Matthews, 2015). A systematic review on retirement and health suggests retirement has a beneficial effect on mental health (van der Heide, van Rijn, Robroek, Burdorf, & Proper, 2013), although meta-analyses from the same review suggest that retirement has no overall effect on perceived general health and physical health.

There is also some evidence that the health benefits of retirement mainly occurs among employees working in low occupational grades and poor quality work (Westerlund et al., 2009), who report improved health on retirement, although the improvements were only observed for self-reported and mental health measures and not for physical health (Westerlund et al., 2010). The improvement in mental health shortly after retirement may be a consequence of a reduction in work-related stressors, particularly for those in the lowest occupational grades and poorest working conditions. There is evidence that chronic work stressors are more prevalent in the lowest occupational grades (Wahrendorf, Dragano, & Siegrist, 2013), as well as evidence that such chronic stressors affect physical (Chandola, Brunner, & Marmot, 2006) and mental health (Stansfeld & Candy, 2006). However, the retirement-related improvement in health for employees in low occupational grades and poor quality work appears to contradict other observed patterns of widening social inequalities in health in early old age and retirement.

One of the limitations of existing studies on retirement and health is a lack of studies on related biological processes stress is often inferred from mental health measures, but direct biophysiological measures associated with the stress response are often not measured. There is, as yet, no evidence on a reduction in physiological stress levels around retirement, particularly for those in low occupational grades and poor working conditions. It may be useful to examine what happens to biophysiological measures associated with stress around the period of retirement, as self-reported measures of stress and mental health may be confounded by social status and perceptions of health. With retirement and pension age increasing in most countries, there is a need to examine whether such policies could impact on the wellbeing of employees in disadvantaged working conditions.

There is now considerable scientific evidence linking lower and more disadvantaged social positions to higher levels of biomarkers of stress (Dowd, Simanek, & Aiello, 2009; Kumari et al., 2010a; Kunz-Ebrecht, Kirschbaum, & Steptoe, 2004; Matthews & Gallo, 2011; Steptoe et al., 2003). Cortisol is a stress hormone that follows a diurnal profile, peaking around 30 minutes after awakening, and returning to very low levels by bedtime. Stress disrupts the diurnal profile of cortisol, resulting in elevated levels of cortisol and a flatter diurnal slope from the awakening response to bedtime. Flatter diurnal cortisol slopes are thus a key biomarker associated with stress, indicating dysregulation in the endocrine stress response system (Adam & Kumari, 2009). Flatter diurnal cortisol slopes are also associated with cardiovascular mortality—a 1 SD increase in cortisol at bedtime was associated with a doubling of the relative risk of cardiovascular mortality within 6–8 years (Kumari, Shipley, Stafford, & Kivimaki, 2011). Previous analyses of diurnal cortisol slopes in the same study found two distinct patterns of diurnal cortisol secretion—a “normative” versus a “raised” pattern with the latter characterized by higher diurnal cortisol and a flatter diurnal slope, which in turn was associated with older age, smoking status, stress on the day of sampling and shorter sleep duration (Kumari et al., 2010b).

However, a major limitation of previous studies on social position and cortisol is a lack of longitudinal analyses, especially in relation to changes in social position and stress. There are different theories about how a change in social position affects stress and the associated biological response. The impact of social position on stress may differ by stability of social rank. The physiological and psychological advantages of high social rank disappears when rank is unstable among baboons (Sapolsky, 1992). Among humans, the period around retirement could be particularly important as status based on occupation rankings could change during the transition to retirement. A steep upward career path in the life-history of Dutch retirees was associated with more difficulties adjusting to the loss of status following retirement (Damman, Henkens, & Kalmijn, 2015). Furthermore, as work-related stressors are more prevalent among lower ranking occupations, retirement is likely to reduce stress among low-grade employees. Thus, the social gradient in stress may become less pronounced as employees transition into retirement because of a reduction in work-related stress among employees in lower ranking occupations.

A further limitation of existing cross-sectional research on socioeconomic differences in stress is the lack of controls for previous levels of stress and health. Employees who are stressed or more unhealthy are also more likely to leave their jobs (Wahrendorf et al., 2013), resulting in a “survivor” workforce who are better able to cope with stressors or who are less stressed. It is thus difficult to compare the cross-sectional stress levels of employees who survive in employment to their “retired” peers, as they may have been differentially exposed to work stressors, or those who remain in employment may be more resilient to stressors and ill-health.

This study investigated whether a recent transition into retirement was associated with steeper (more advantageous) diurnal cortisol slopes compared to those in employment in later life. Furthermore, we examined whether retirement was associated with steeper diurnal slopes for those at the bottom of the occupational hierarchy who transitioned into retirement, compared to their peers who remained in employment.

## Methods

### Study Participants

The data analysed were from the 7^th^ (2002–2004), 8th (2006), and 9^th^ (2007–09) phases of the Whitehall II cohort study. The cohort was initially recruited between 1985 and 1988 (phase 1) from 20 London-based civil service departments. Eleven phases of the study have been completed, although only phases 7 and 9 have saliva samples for the assessment of cortisol. The initial cohort (of 10,308 employees) was recruited between 1985 and 1988 from 20 London-based civil service departments (Marmot & Brunner, 2005). Ethical approval for the Whitehall II study was obtained from the University College London Medical School committee on the ethics of human research. Informed consent was gained from every participant.

As the research questions focus on recent transitions from employment to retirement, transitions among those who were in employment at phase 8 (2006) and who were either still in work or self-reported as retired by phase 9 (2007–09) were analysed (*n* = 2,598). Among them, only 1,501 had cortisol measured at phase 7, and a further 1,190 had cortisol measured at phase 9. The analytical sample further reduced (*n* = 1,143) with missing covariate data.

### Cortisol Collection and Analysis

Respondents provided (by post) six saliva samples in salivettes over the course of a normal (working) weekday at waking, +30 mins, +2.5 hours, +8 hours, +12 hours, and bedtime at phases 7 and 9. Only a subsample of all phase 7 respondents could participate because of a late start to the data collection for cortisol, but all phase 9 respondents were eligible for cortisol data collection. Participation rates in cortisol collection were around 90% at both phases. Respondents used a log book to record information on their waking time and the time each sample was taken; technical details are available here (Kumari, Shipley, Stafford, & Kivimaki, 2011). There was a right skewed distribution of cortisol values and cortisol data were transformed by natural logarithm for the regression analysis.

### Exposures

#### Hours since awakening

This was based on respondents recording of waking time and time of sampling in the log book diary. Linear and quadratic terms of the time variable were used in the models to estimate nonlinear effects.

#### Employment grade

Civil service employment grade was used to categorize people into high, middle, or low grades. Civil service jobs are predominantly nonmanual, although a few office support staff were messengers and porters (Marmot & Brunner, 2005). About half of those still employed at phase 8 were still working in the civil service. Those employed elsewhere were classed according to their last civil service grade as that is a strong predictor of their health, and similar to the low prevalence of manual jobs in the civil service, very few of ex-civil servants (<5%) were working in partly skilled or unskilled manual jobs.

All participants were working at baseline (phase 8). Employment status at follow up (phase 9) was self-reported. Only those who remained in employment or had stated they had retired were included in the analysis. Those who were unemployed (*n* = 32), too sick to work (*n* = 4), or were looking after their family (*n* = 12) were not included in the analysis as these other labor market and economic inactivity statuses are likely to be associated with poorer health. Due to the healthy worker effect (Li & Sung, 1999), we tried to keep the control (still employed) and treatment (retired) groups as similar as possible by removing from the analysis those workers who had stopped working for any other reason apart from self-reporting as retired.

### Covariates

Age, sex, and smoking status were assessed by questionnaire. Current smokers were defined as those who reported smoking cigarettes, cigars or a pipe, social or occasional smoking, or taking nicotine replacement products. Body mass index (BMI) groups (<20, 20–25, 25–30, and 30+) were derived from height and weight measurements by trained nurses at phases 7 and 9. Hours slept the night before day of saliva sample collection was based on the respondents recording in the log book diary of their time of falling asleep and their awakening time. Sleep duration was grouped into 5 categories (<5 hours, 5–6 hours, 6–7 hours, 7–8 hours, and 8 hours+). Self-reported health was measured by a question on general health (5-point Likert scale going from excellent to poor) and the report of any long standing illness. Marital status was categorized into never married, currently married/cohabiting, and formerly married/cohabiting. All the continuous covariates (age, BMI, sleep hours, and awakening time) were categorized to facilitate the interpretation of the interactions with the continuous hours since awakening variable.

### Statistical Methods

As the cortisol values are sampled across the day and clustered within individual respondents, multilevel growth curve models were analysed. These models account for the clustering of sampled occasions of cortisol collection within individuals, as well as between-individual heterogeneity in the diurnal slopes, by estimating random slopes for the hours since awakening variable. Only a random slope for the linear hours since awakening variable was estimated as the random slope for the quadratic term resulted in very small estimates and negative variance estimates.

In each of these models, the phase 9 log cortisol values on awakening, at 2.5, 8, and 12 hours later and at bedtime were regressed on hours since awakening, the employment grade/status variables and the other covariates. The second diurnal cortisol sample at 30 minutes after awakening was not included as the cortisol awakening response and the diurnal slope may have differing biological underpinnings (Adam & Kumari, 2009).

The interaction terms between the employment grade, employment status and hours since awakening were included in the models to test whether the diurnal cortisol slopes differed between employment grade and status categories. These interaction terms between these covariates and hours since awakening were added to the model even if the main effects of the covariates were not statistically significant, as the nonsignificant average diurnal effect of the covariates could be masking significant differences between the diurnal slopes. This could occur due to “qualitative” or “crossover” interactions (VanderWeele & Knol, 2014). Statistical significance at the 5% level was measured by the change in Deviance (−2 * Log Likelihood Ratio) of nested models. All the models controlled for key confounders measured at phase 9, that have previously been shown to be associated with diurnal cortisol—age, sex, smoking status, BMI, sleep hours, and awakening time, as well as the significant interactions of each of these covariates with hours since awakening. We also controlled for the phase 7 values of log cortisol and the interaction with hours since awakening, which results in the interpretation of regression coefficients as change in cortisol slopes (from phase 7 to 9). However, the addition of the Wave 7 diurnal cortisol variables to the models made very little difference to the other coefficients in the model, with the exception of the intercept (see Supplementary Appendix Table 2 for a comparison of final model coefficients from Table 2 with and without Wave 7 diurnal cortisol). Hence, rather than describing the coefficients in Table 2 in terms of change in diurnal cortisol slopes, we have ignored interpreting the effect of lagged cortisol, and instead interpreted the coefficients in terms of associations with the phase 9 diurnal cortisol slopes.

We additionally tested whether other potential confounders such as marital status (at phase 9), and other potential selection factors such as self-reported health and longstanding illness (measured at phase 7, as these are potential selection factors), and their interactions with hours since awakening were associated with diurnal cortisol and found no evidence for improvement in model fit, so these were removed from further analysis. All the multilevel analyses were carried out using MLwin software.

## Results

Table 1 displays the mean cortisol on awakening and bedtime at phases 7 and 9 of the Whitehall II study by key explanatory variables in the analysis. Employees working in the lowest grades had lower cortisol values on awakening but higher cortisol at bedtime in both periods. This suggests that the low-grade employees had flatter diurnal slopes at both phases. A similar pattern of flatter diurnal slopes was observed for the employed (compared to the retired) at both phases. At phase 9, flatter slopes were observed for participants reporting short sleep hours (<6 hours). The mean age of employees at phase 8 was 59.9 years (with a standard deviation of 4.2). By phase 9, the mean age of those who retired was 62.9, and among those still in employment was 61.7 years.

**Table 1.** Mean (95% Confidence Intervals) of Cortisol Upon Awakening and at Bed Time by Levels of Key Explanatory Variables, Whitehall II: Phases 7 (2002–04) and 9 (2007–09)

	2002–04 Cortisol upon awakening	2002–04 Cortisol at bed time	*n*	2007–09 Cortisol upon awakening	2007–09 Cortisol at bed time	*n*
Civil service grade (2007–09)						
High	17.72 [16.29, 19.14]	2.82 [2.40, 3.24]	576	15.72 [15.10, 16.33]	2.61 [2.33, 2.88]	576
Middle	16.73 [15.82, 17.65]	2.26 [2.02, 2.49]	500	14.94 [14.25, 15.63]	3.38 [2.76, 4.01]	500
Low	15.26 [13.12, 17.40]	2.89 [1.98, 3.79]	67	14.43 [12.31, 16.54]	4.02 [2.63, 5.40]	67
Employment status (2007–09)						
Employed	16.75 [15.74, 17.77]	2.32 [1.99, 2.65]	280	15.30 [14.34, 16.27]	3.08 [2.18, 3.97]	280
Retired	17.27 [16.22, 18.32]	2.66 [2.36, 2.96]	863	15.30 [14.79, 15.81]	3.01 [2.71, 3.32]	863
Age-group (2007–09)						
55–60	16.96 [15.89, 18.03]	2.26 [2.01, 2.51]	437	14.98 [14.29, 15.66]	2.69 [2.37, 3.00]	437
60–65	17.64 [16.00, 19.27]	2.73 [2.31, 3.14]	479	15.50 [14.75, 16.25]	3.14 [2.65, 3.64]	479
65–70	16.94 [15.44, 18.43]	3.01 [2.00, 4.02]	149	15.61 [14.35, 16.87]	3.42 [1.96, 4.88]	149
70–79	15.53 [13.93, 17.13]	2.62 [2.08, 3.17]	78	15.30 [13.81, 16.80]	3.50 [2.21, 4.80]	78
Gender						
Men	17.04 [16.26, 17.81]	2.61 [2.33, 2.90]	904	15.52 [15.00, 16.04]	2.98 [2.61, 3.34]	904
Women	17.54 [14.86, 20.22]	2.44 [2.00, 2.88]	239	14.48 [13.57, 15.40]	3.23 [2.59, 3.87]	239
Sleep hours (wave specific)						
<6 hours	17.13 [13.99, 20.27]	2.98 [2.41, 3.55]	210	14.33 [13.05, 15.62]	3.94 [2.98, 4.91]	148
6–7 hours	17.17 [15.79, 18.54]	2.57 [2.07, 3.08]	366	15.39 [14.58, 16.20]	2.77 [2.45, 3.09]	345
7–8 hours	17.03 [16.02, 18.04]	2.37 [2.03, 2.72]	390	15.37 [14.63, 16.11]	2.70 [2.32, 3.07]	409
>8 hours	16.75 [15.52, 17.98]	2.34 [1.80, 2.88]	141	15.65 [14.63, 16.67]	3.40 [2.26, 4.54]	241

Table 2 displays the results of the multilevel model with the log transformed salivary cortisol sampled throughout the day as the dependent variable. All the models were adjusted for age-group, employment status, gender, sleep hours, awakening time, smoking status, and Wave 7 diurnal cortisol levels, as well as their interactions with the hours since awakening variable. BMI, marital status, general health, longstanding illness, and their interactions with hours since awakening did not improve model fit and so were excluded from further analysis. Similarly, the interaction of gender with hours since awakening was not significant so gender was only a main effect in the displayed models. The additional covariates from all the models are displayed in Supplementary Appendix Table 1.

**Table 2.** Estimates (95% CI) From Multilevel (Occasions Within Individuals) Models of Log Diurnal Cortisol at Phase 9 of the Whitehall II Study

Fixed part	Model 1	Model 3	Model 5	Model 6	Model 7
Intercept: log cortisol on awakening	1.99 (1.83, 2.15)	2.03 (1.86, 2.20)	2.03 (1.84, 2.21)	1.99 (1.79, 2.18)	2.00 (1.80, 2.19)
Hours since awakening (linear)	−0.10 (−0.12, −0.07)	−0.11 (−0.13, −0.08)	−0.11 (−0.14, −0.09)	−0.11 (−0.14, −0.09)	−0.12 (−0.14, −0.09)
Hours since awakening (quadratic)	0.001 (0.001, 0.002)	0.001 (0.001, 0.002)	0.001 (0.001, 0.002)	0.001 (0.001, 0.002)	0.001 (0.001, 0.002)
Occupational grade (ref: high grade)					
Middle grade		−0.04 (−0.11, 0.02)	−0.04 (−0.11, 0.02)	0.02 (−0.10, 0.13)	−0.02 (−0.15, 0.12)
Low grade		−0.13 (−0.27, 0.01)	−0.13 (−0.27, 0.01)	0.08 (−0.13, 0.29)	0.11 (−0.13, 0.35)
Interaction of occupational grade and hours since awakening (ref: high grade on awakening)					
Middle grade * hours since awakening		0.01 (0.005, 0.02)	0.01 (0.005, 0.02)	0.01 (0.005, 0.02)	0.02 (0.004, 0.03)
Low grade * hours since awakening		0.02 (0.01, 0.04)	0.03 (0.01, 0.04)	0.03 (0.01, 0.04)	0.02 (−0.01, 0.05)
Employment status (ref: retired)					
Still in employment at phase 9			0.003 (−0.07, 0.08)	0.06 (−0.04, 0.16)	0.04 (−0.06, 0.15)
Interaction of employment status and hours since awakening (ref: retired on awakening)					
Still in employment at phase 9 * hours since awakening			0.01 (0.001, 0.02)	0.01 (0.001, 0.02)	0.01 (−0.004, 0.02)
Interaction of occupational grade and employment status (ref: high grade or retired at phase 9)					
Middle grade and employed at phase 9				−0.08 (−0.20, 0.05)	−0.03 (−0.18, 0.12)
Low grade and employed at phase 9				−0.31 (−0.55, −0.07)	−0.36 (−0.65, −0.07)
Interaction of occupational grade, employment status, and hours since awakening (ref: high grade or retired at phase 9 on awakening)					
Middle grade and employed at phase 9 * hours since awakening					−0.01 (−0.02, 0.01)
Low grade and employed at phase 9 * hours since awakening					0.01 (−0.02, 0.04)
Random part					
Individual level intercept	0.037	0.036	0.036	0.035	0.035
Individual level slope (linear hours)	0.001	0.001	0.001	0.001	0.001
Covariance: individual intercept and slope	0.002	0.002	0.002	0.002	0.002
Occasion level intercept	0.446	0.446	0.446	0.446	0.446
Deviance (−2 * loglikelihood)	12762.23	12744.43	12738.30	12731.43	12729.69
*p* value for change in deviance		0.001	0.047	0.032	0.420

*Note:* CI, confidence interval.

Table 2 displays coefficients that are the same for all individuals (“fixed”) and that differ between individuals (“random”) from the different multilevel models. As we are interested in comparing the diurnal cortisol slopes of different individuals and groups, we first needed to describe the “growth curves” of diurnal cortisol (Model 1). A standard growth curve model has a (fixed) time coefficient that is the average increase or decrease in the dependent variable per unit of time. In addition, a random slope of the time coefficient was estimated to allow for differing growth rates for individuals.

Model 1 examined the baseline model with the linear and quadratic terms for hours since awakening as the key explanatory variables, adjusted for all the covariates mentioned above. The negative linear hours since awakening coefficient (or “slope”) is the average diurnal decline in cortisol (measured in log nmol/l) per hour, which is moderated by the positive quadratic hours term later in the day. An additional hour after awakening was associated with an average reduction of 0.1 of log cortisol, although there was some variation between individuals in these diurnal cortisol slopes (indicated by the random terms for the linear slope). Model 2 (not shown in the Table) added in employment grade, but the coefficients for middle and low grades overlapped zero and there was no improvement in model fit. Despite the lack of evidence of a main effect of employment grade, the interaction of grade with hours since awakening was entered into Model 3 (due to a potential “crossover interaction” as mentioned in the Methods section) and this resulted in an improvement in model fit. The coefficients for grade (which were close to zero in Model 2) became more negative, indicating that the lower grade employees tended to have lower cortisol on awakening. Furthermore, the interaction terms with hours since awakening were positive, indicating that the lower grade employees tended to have flatter diurnal cortisol slopes. Model 4 (not shown in the Table) added in employment status and Model 5 (shown in Table 2) added in the interaction between employment status and hours since awakening. Employees still in employment at phase 9 had similar levels of cortisol when they woke up compared to their peers who retired. However, the diurnal decline in cortisol was flatter (+0.01 log cortisol per hour) for those remaining in employment compared to those who retired, indicating that retirement was associated with steeper diurnal cortisol slopes. Model 6 added in the interaction of employment grade and status, which further improved the fit of the model, although the three-way interaction between employment status, grade, and hours since awakening (Model 7) did not improve model fit.

**Figure 1.**
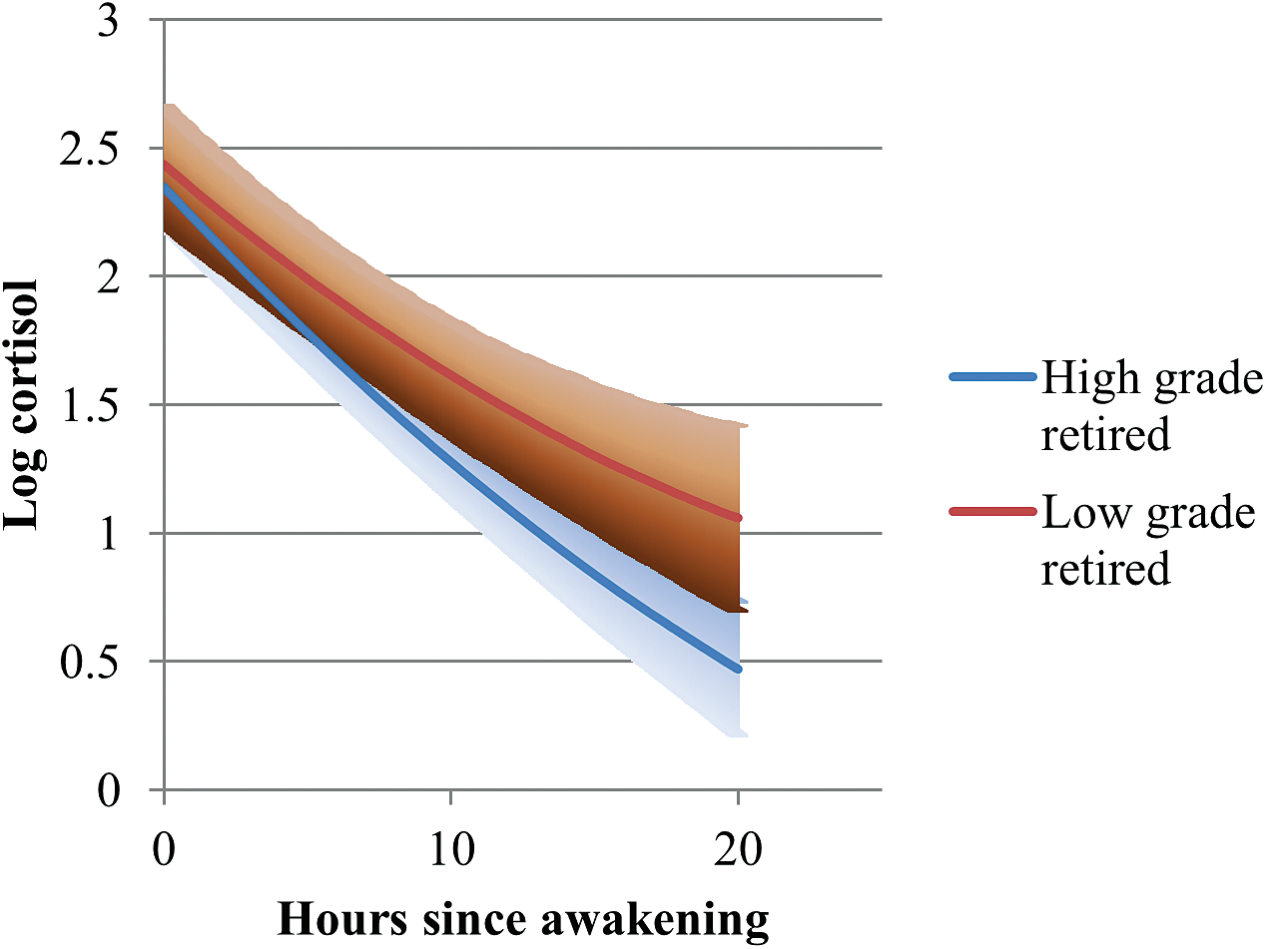
Diurnal cortisol slope estimates (and 95% CI): high versus low occupational grade, RETIRED respondents. CI = confidence interval.

**Figure 2.**
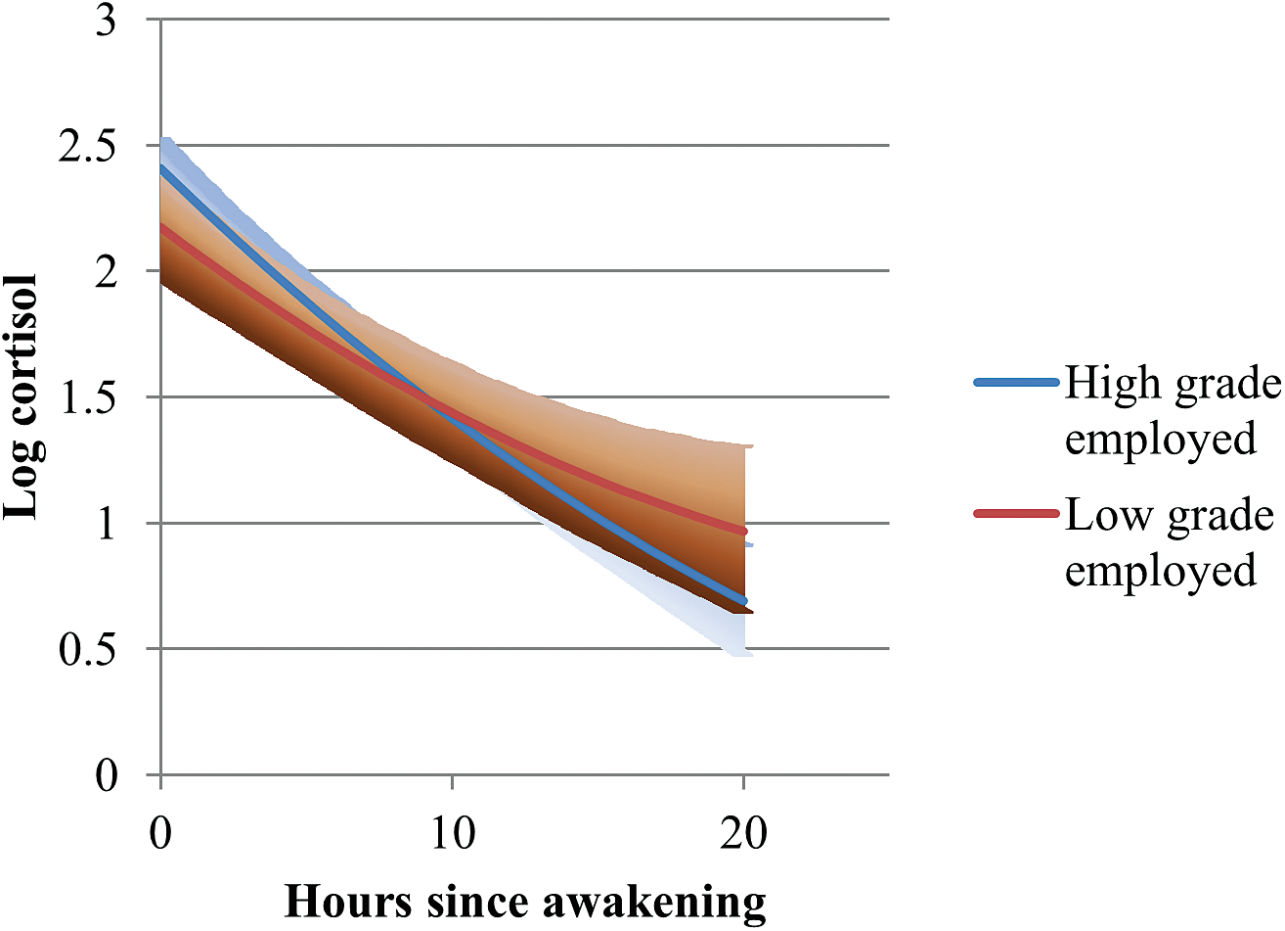
Diurnal cortisol slope estimates (and 95% CI): high versus low occupational grade, EMPLOYED respondents. CI = confidence interval.

**Figure 3.**
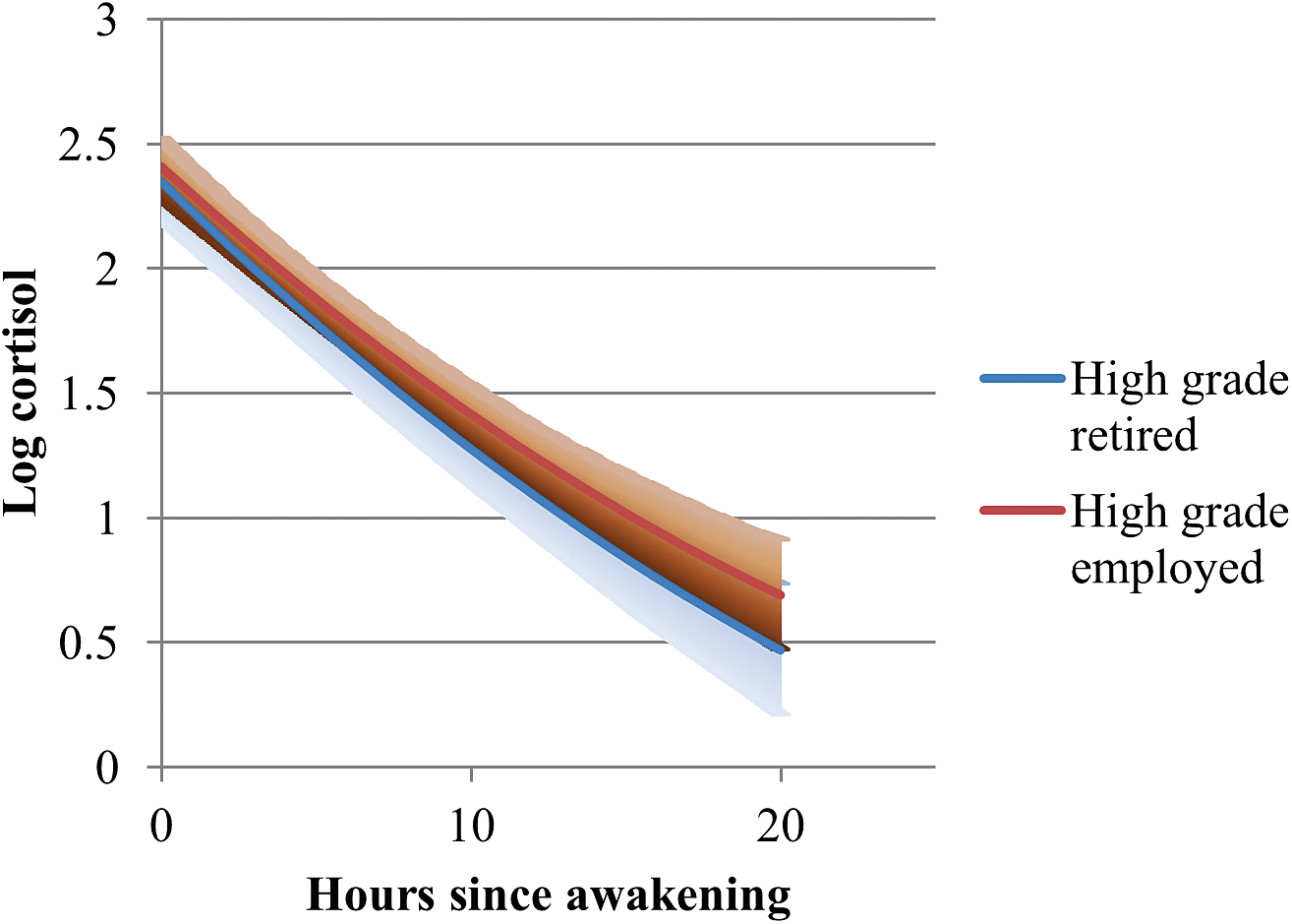
Diurnal cortisol slope estimates (and 95% CI): retired versus rmployed, HIGH grade respondents. CI = confidence interval.

**Figure 4.**
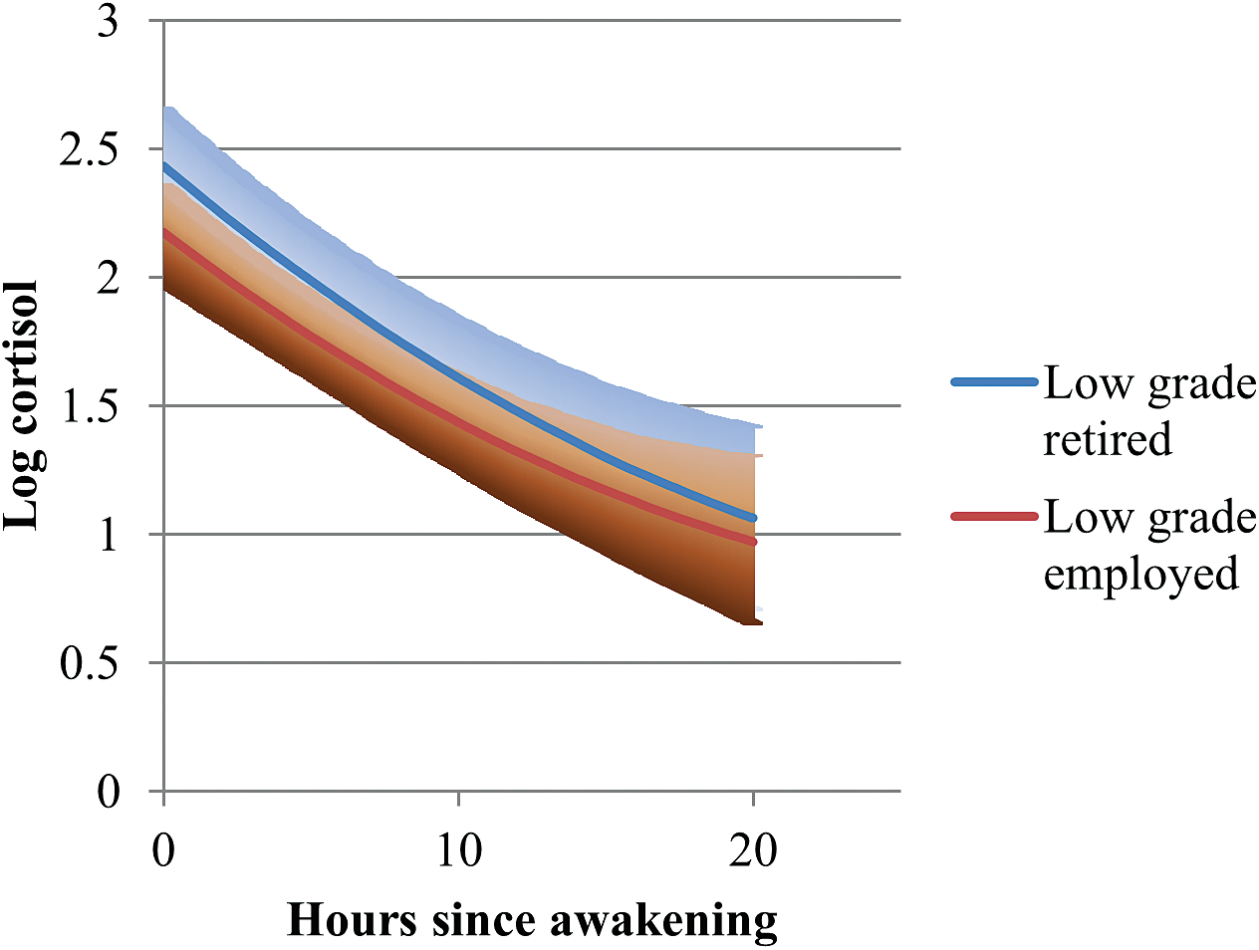
Diurnal cortisol slope estimates (and 95% CI): retired versus employed, LOW grade respondents. CI = confidence interval.

As the coefficients for interaction terms in Model 6 are hard to interpret in isolation given the other interactions in the model, specific interaction effects are presented in Figures 1–4. The diurnal slopes for all three occupational groups are hard to distinguish when plotted in the same figure, so only the estimated diurnal slopes for the high- and low-grade employees are shown in the figures, although all three occupational groups (including middle-grade employees) were included in the interaction analyses (see Table 2, Models 4–7). Among respondents who had recently retired, (Figure 1), there was a clear occupational difference in the diurnal cortisol slope—employees who were formerly in the lowest grades have flatter slopes and higher diurnal cortisol levels by bedtime compared to those who were formerly employed in the high grades. The 95% confidence intervals for the low-grade retirees overlapped with the cortisol estimates for the high-grade retirees on awakening. But as the day went on, the lower confidence intervals for the low-grade retirees were distinctly higher than the cortisol estimates for the high-grade retirees. Among participants still in employment at phase 9 (Figure 2), although the estimated slopes for low-grade employees were flatter than for high-grade employees, they were not distinct from each other, especially toward evening and night time. Another way of examining the interaction is by looking at the employment status differences within occupational grades (Figures 3 and 4). Among the high-grade employees, those who had retired had steeper estimated diurnal slopes compared to their peers who remained in employment (Figure 3). The lower 95% confidence intervals of higher cortisol later in the day for high-grade employees who were still working at phase 9 did not overlap with the estimated cortisol for their peers who had retired. Comparing the lowest grade employees, there was not much difference in the slopes of those who retired and those still in employment (Figure 4).

## Discussion

This study has shown that British civil servants employed in the lowest occupational grades had flatter (more adverse) diurnal cortisol slopes compared to those in the highest grades. The occupational gradient in diurnal cortisol slopes was distinct among those who had recently retired, but less clear among those who were still working past standard retirement age (60 years for this cohort). Retirement was associated with steeper (more advantageous) diurnal slopes compared to those who remained in work; however, there was no difference in the retirement slope by occupational grade. Retirement was not associated with more advantageous cortisol profiles for those formerly employed in the lowest grades in comparison to their peers who were still working.

This study thus appears to find some evidence that the occupational gradient in diurnal cortisol is different for retired compared to employed British civil servants working past standard retirement age. Rather than retirement being associated with lower levels of biomarkers of stress for those at the bottom of the occupational hierarchy, this period of the lifecourse appears to reinforce existing inequalities and widen the gap between occupational groups. The hypotheses were based on previous evidence suggesting that the period around retirement is associated with an improvement in self-rated health among employees in poor quality and low-grade jobs around retirement in the GAZEL study (Westerlund et al., 2009). This discrepancy in findings may be driven by the different populations in the two studies. The GAZEL study included blue collar workers, with physically demanding jobs, with potentially higher stress levels compared to British civil servants. Furthermore, the follow up period for this study (2–3 years) was longer than the 1-year measurement intervals of the 15-year follow up in GAZEL. It is possible that any effects of retirement on improving the stress levels of employees in the lowest grades may only be temporary and not have an effect a year or more after retirement. Moreover, the measures of self-rated health used in the GAZEL study may not correspond to levels of cortisol used in this study.

Although this study did not directly examine the role of work stress on diurnal cortisol profiles, previous research from the Whitehall II study has shown that effort-reward imbalance was associated with flatter diurnal cortisol profiles (Liao, Brunner, & Kumari, 2013). Effort-reward imbalance was lower among higher grade occupations (Wahrendorf et al., 2013), so the removal of work stressors through retirement is unlikely to explain the improved cortisol profiles of those recently retired from the highest grades. A previous report from the Whitehall II study also found an improvement in mental health for employees working in the highest grades when they retire, with no corresponding improvement among employees working in the lowest grades (Mein, Martikainen, Hemingway, Stansfeld, & Marmot, 2003). Civil servants working in the highest grades would normally receive higher pensions and may be more able to enjoy the leisure and social participation and activities in retirement due to their higher levels of resources and better health than those retiring from low-grade occupations. Qualitative interviews among lower grade civil servants suggest that they worry about their reduced income in retirement (Mein, Higgs, Ferrie, & Stansfeld, 1998).

Respondents self-reported their retirement status, which could lead to measurement bias as many “retired” people still engage in paid employment (Banks, Chandola, & Matthews, 2015). We did not collect data on reasons for retirement for all participants, although early exit due to health reasons is unlikely to be a key reason as the mean age of participants at baseline was so close to retirement age and the health measures assessed at baseline did not predict the diurnal cortisol slopes. We relied on self-reports for the timing of cortisol sample collection as evidence suggests that people are usually accurate in reporting this information (Dockray, Bhattacharyya, Molloy, & Steptoe, 2008).

The study did not look at within person change (fixed effects) because the aim was to examine the between person differences (differences between occupational grades). As very few civil servants change employment grades at this age, a fixed-effects model would have deleted anyone with stable characteristics like employment grade, making it impossible to compare employees from different occupational grades. Therefore, we did not estimate a causal effect of transitioning from employment to retirement, although we tried to reduce selection bias by controlling for a number of relevant confounders, including previous levels of cortisol. Additionally, our sample of older workers was on average, aged just under standard retirement age (aged 60 years) at baseline and was followed up for around 2 to 3 years after. The healthy worker effect suggests that our baseline sample were a healthy cohort as they remained in employment close to retirement age. Furthermore, we tried to reduce health-selection effects from the follow up sample by removing from the analysis, those who had stopped working for any other reason apart from self-reporting as retired. This is because stopping working for reasons of limiting health, unemployment, or family issues is likely to be correlated with health and could confound the reported associations. Around 51% of employees were working in noncivil service jobs. Their last civil service grade (prior to leaving the civil service) may not accurately reflect their current occupational position, although very few (<5%) who were working outside the civil service were in partly skilled and unskilled manual work, which reflects the low prevalence of manual work in the civil service. A further limitation is the lack of generalizability of the results to working conditions outside the civil service. However, civil servants tend to have very good working conditions, compared to the general working population. As this study found an association between low-grade work and stress among civil servants, it is likely that such an effect is underestimated, given that poor working conditions are more prevalent in the general working population compared to the civil service. Only respondents with diurnal cortisol data at phases 7 and 9 were included in the analysis, resulting in a loss of 56% of the respondents who were in employment at phase 8, with lower grade employees over-represented. Such a loss in sample size could have biased the results if there were occupational grade by employment status differences in the missing versus nonmissing respondents. Further analysis (not shown) revealed that employees in the lowest civil service grade who were still in employment at phase 9 were most likely to have missing cortisol data. As low-grade employees have flatter diurnal cortisol slopes, the higher prevalence of missing cortisol data among low-grade employees also suggests an underestimation of the reported associations between lower occupational grade and flatter diurnal slopes. The estimated cortisol slopes were based on data collected in 2007–09. Working and retirement conditions for current older employees in the civil service and elsewhere may be different nowadays with some groups of older workers having relatively stable jobs and pension arrangements, and other older workers having more unstable working and retirement conditions.

The analysis of biological markers of stress in relation to transitions into retirement has revealed new insights on the process of retirement, stress, and inequalities. Occupational grade differences in a biomarker of stress were larger among the retired compared to those currently employed. Employees in the highest grades appeared to benefit the most from retirement in terms of steeper diurnal cortisol slopes, whereas there was no apparent benefit of retirement among those working in the lowest grades. These biological differences associated with transitions into retirement for different occupational groups may partly explain the pattern of widening social inequalities in health in early old age. Further research using more representative population samples on the biological processes underlying socioeconomic inequalities in health could help identify important older age lifecourse factors that generate widening health inequalities in early old age.

## Supplementary Material

Supplementary material are available at *The Journals of Gerontology Series B: Psychological and Social Sciences* online.

## Funding

Research for this study was supported by the U.K. Economic and Social Research Council–funded International Centre for Life Course Studies in Society and Health (ES/J019119). The Whitehall II study has been supported by grants from the Medical Research Council; British Heart Foundation; National Heart Lung and Blood Institute (R01HL36310), USA, NIH: National Institute on Aging (R01AG13196 and R01AG34454), USA, NIH.

## Supplementary Material

Appendix Table 1
