# Supplementary material for: Retirement and Socioeconomic Differences in Diurnal Cortisol: Longitudinal Evidence From a Cohort of British Civil Servants
Source: J Gerontol B Psychol Sci Soc Sci. 2017 May 5;73(3):447–56. doi: 10.1093/geronb/gbx058 (PMC5927089; doi:10.1093/geronb/gbx058)
Supplement: Appendix Table 1 [file gbx058_suppl_appendix_table_1.docx]

**Appendix Table 1: Additional covariates adjusted for in Models 1-6 from Table 2**

| **Fixed Part** | Model 1 | Model 3 | Model 5 | Model 6 |
| --- | --- | --- | --- | --- |
| *Age-group (ref: 55-59)* |  |  |  |  |
| age 60-64 | -0.023 (-0.09,0.05) | -0.021 (-0.09,0.05) | -0.021 (-0.09,0.05) | -0.021 (-0.09,0.05) |
| age65-69 | -0.03 (-0.13,0.07) | -0.04 (-0.14,0.06) | -0.04 (-0.13,0.06) | -0.04 (-0.13,0.06) |
| age 70+ | 0.01 (-0.12,0.14) | 0.01 (-0.12,0.14) | 0.02 (-0.11,0.14) | 0.02 (-0.11,0.14) |
| *Age*Hrs after awaken.* |  |  |  |  |
| age 60-64*Hours since awakening | 0.004 (-0.004,0.01) | 0.004 (-0.004,0.01) | 0.005 (-0.003,0.01) | 0.005 (-0.003,0.01) |
| age 65-69*Hours since awakening | 0.01 (0.003,0.03) | 0.02 (0.004,0.03) | 0.02 (0.01,0.03) | 0.02 (0.01,0.03) |
| age 70+*Hours since awakening | 0.01 (-0.002,0.03) | 0.01 (-0.002,0.03) | 0.01 (-0.0001,0.03) | 0.01 (-0.001,0.03) |
| *Men (ref: Women)* | 0.06 (-0.01,0.12) | 0.06 (-0.01,0.12) | 0.06 (-0.01,0.12) | 0.06 (-0.01,0.12) |
| *Current smoker (ref: not a current smoker)* | -0.08 (-0.22,0.05) | -0.07 (-0.21,0.07) | -0.071 (-0.21,0.07) | -0.071 (-0.21,0.07) |
| *Smoking*Hrs after awaken.* | 0.01 (-0.002,0.03) | 0.01 (-0.01,0.03) | 0.01 (-0.01,0.03) | 0.01 (-0.01,0.03) |
| *Hours slept (ref: <5 hours)* |  |  |  |  |
| Slept 5-6 hours | 0.025 (-0.08,0.13) | 0.02 (-0.08,0.13) | 0.03 (-0.08,0.13) | 0.03 (-0.08,0.13) |
| Slept 6-7 hours | 0.02 (-0.09,0.13) | 0.02 (-0.09,0.13) | 0.02 (-0.09,0.13) | 0.02 (-0.09,0.13) |
| Slept 8+ hours | 0.05 (-0.07,0.18) | 0.05 (-0.08,0.17) | 0.05 (-0.08,0.17) | 0.05 (-0.08,0.17) |
| *Hours slept*Hrs after awaken.* |  |  |  |  |
| Slept 5-6 hours*Hours since awakening | -0.01 (-0.02,-0.002) | -0.01 (-0.02,-0.002) | -0.01 (-0.02,-0.002) | -0.01 (-0.02,-0.002) |
| Slept 6-7 hours*Hours since awakening | -0.02 (-0.03,-0.004) | -0.02 (-0.03,-0.004) | -0.02 (-0.03,-0.004) | -0.02 (-0.03,-0.004) |
| Slept 8+ hours*Hours since awakening | -0.02 (-0.04,-0.01) | -0.02 (-0.04,-0.01) | -0.02 (-0.04,-0.01) | -0.02 (-0.04,-0.01) |
| *Awakening Time (ref: awoke before 6am)* |  |  |  |  |
| Awoke 6-7am | 0.04 (-0.06,0.13) | 0.03 (-0.07,0.12) | 0.03 (-0.07,0.12) | 0.03 (-0.07,0.12) |
| Awoke 7-8am | -0.04 (-0.14,0.06) | -0.05 (-0.15,0.05) | -0.05 (-0.15,0.05) | -0.05 (-0.15,0.05) |
| Awoke after 8am | -0.15 (-0.28,-0.02) | -0.15 (-0.28,-0.03) | -0.15 (-0.28,-0.03) | -0.15 (-0.28,-0.03) |
| *Awakening Hours*Hrs after awaken.* |  |  |  |  |
| Awoke 6-7am*Hours since awakening | -0.01 (-0.02,0.001) | -0.01 (-0.02,0.004) | -0.01 (-0.02,0.004) | -0.01 (-0.02,0.004) |
| Awoke 7-8am*Hours since awakening | -0.01 (-0.02,0.001) | -0.01 (-0.02,0.004) | -0.01 (-0.02,0.004) | -0.01 (-0.02,0.004) |
| Awoke after 8am*Hours since awakening | 0.003 (-0.01,0.02) | 0.004 (-0.01,0.02) | 0.01 (-0.01,0.02) | 0.01 (-0.01,0.02) |
| *Wave 7 log cortisol levels* | 0.19 (0.15,0.24) | 0.19 (0.15,0.23) | 0.19 (0.15,0.23) | 0.19 (0.15,0.23) |
| *wave 7 log cortisol levels*Hrs after awaken.* | -0.004 (-0.01,-0.008) | -0.004 (-0.01,-0.008) | -0.004 (-0.01,-0.008) | -0.004 (-0.01,-0.008) |

Appendix Table 2: Comparison of coefficients from Model 6 (Table 2, manuscript) with the same model without the lagged wave 7 cortisol values

| **Fixed Part** | Model 6 (from Table 2) | Model 6 without the wave 7 cortisol |
| --- | --- | --- |
| *Intercept: Log cortisol on awakening* | 1.99 (0.10) | 2.49 (0.08) |
| *Slope or Hours since awakening (linear term)* | -0.11 (0.01) | -0.15 (0.01) |
| *Hours since awakening (quadratic term)* | 0.001 (0.0004) | 0.002 (0.0003) |
| *Occupational Grade (ref: High Grade)* |  |  |
| Middle Grade | 0.02 (0.06) | 0.01 (0.06) |
| Low Grade | 0.08 (0.11) | 0.04 (0.11) |
| *Occupational Grade*Hours since awakening (ref: High Grade on awakening)* | |  |
| Middle Grade*Hours since awakening | 0.01 (0.004) | 0.01 (0.004) |
| Low Grade*Hours since awakening | 0.03 (0.01) | 0.03 (0.01) |
| *Employment status (ref: retired at phase 9)* |  |  |
| Still in employment at phase 9 | 0.06 (0.05) | 0.06 (0.05) |
| *Employment Status*Hours since awakening (ref: Retired on awakening)* | |  |
| Still in employment at phase 9*Hours since awakening | 0.01 (0.004) | 0.01 (0.004) |
| *Occupational Grade*Employment Status (ref: High Grade or retired at phase 9)* | |  |
| Middle Grade & employed at phase 9 | -0.08 (0.06) | -0.08 (0.06) |
| Low Grade & employed at phase 9 | -0.31 (0.12) | -0.30 (0.12) |
| **Random Part (Variances and covariances)** |  |  |
| *Level: Individual* |  |  |
| Intercept | 0.04 | 0.04 |
| Linear slope (hours since awakening) | 0.001 | 0.001 |
| Intercept-linear slope covariance | 0.002 | 0.002 |
| *Level: occasion* | 0.446 | 0.454 |
| Intercept |  |  |
| Deviance (-2*loglikelihood) | 12731.426 | 12875.406 |
